# Leaf litter identity rather than diversity shapes microbial functions and microarthropod abundance in tropical montane rainforests

**DOI:** 10.1002/ece3.7208

**Published:** 2021-01-29

**Authors:** Laura M. Sánchez‐Galindo, Dorothee Sandmann, Franca Marian, Valentyna Krashevska, Mark Maraun, Stefan Scheu

**Affiliations:** ^1^ JFB Institute of Zoology and Anthropology University of Göttingen Göttingen Germany; ^2^ Centre of Biodiversity and Sustainable Land Use University of Göttingen Göttingen Germany

**Keywords:** Acari, Collembola, decomposition, litter quality, litterbags, metabolic quotient, microorganisms

## Abstract

In tropical forest ecosystems leaf litter from a large variety of species enters the decomposer system, however, the impact of leaf litter diversity on the abundance and activity of soil organisms during decomposition is little known. We investigated the effect of leaf litter diversity and identity on microbial functions and the abundance of microarthropods in Ecuadorian tropical montane rainforests. We used litterbags filled with leaves of six native tree species (*Cecropia andina*, *Dictyocaryum lamarckianum*, *Myrcia pubescens*, *Cavendishia zamorensis*, *Graffenrieda emarginata*, and *Clusia* spp.) and incubated monocultures and all possible two‐ and four‐species combinations in the field for 6 and 12 months. Mass loss, microbial biomass, basal respiration, metabolic quotient, and the slope of microbial growth after glucose addition, as well as the abundance of microarthropods (Acari and Collembola), were measured at both sampling dates. Leaf litter diversity significantly increased mass loss after 6 months of exposure, but reduced microbial biomass after 12 months of exposure. Leaf litter species identity significantly changed both microbial activity and microarthropod abundance with species of high quality (low C‐to‐N ratio), such as *C. andina*, improving resource quality as indicated by lower metabolic quotient and higher abundance of microarthropods. Nonetheless, species of low quality, such as *Clusia* spp., also increased the abundance of Oribatida suggesting that leaf litter chemical composition alone is insufficient to explain variation in the abundances of soil microarthropods. Overall, the results provide evidence that decomposition and microbial biomass in litter respond to leaf litter diversity as well as litter identity (chemical and physical characteristics), while microarthropods respond only to litter identity but not litter diversity.

## Graphical abstract

Leaf litter identity functions as a major driver of the abundance and activity of soil organisms in tropical montane rainforests.

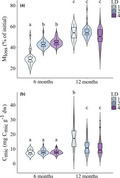

## 1 INTRODUCTION

The great majority of plant material enters the soil as litter, in the form of leaves, stems, and roots. Decomposition of these materials is an essential process for nutrient cycling and provides the basal resources of the soil food web (Berg et al., 1993; Berg & McClaugherty, 2008). In addition to providing food resources, leaf litter accumulating on the soil surface forms a variety of microhabitats for soil organisms, with more diverse litter materials increasing habitat variability, but also providing the opportunity for enhanced nutrient acquisition (Bardgett, 2005; Gessner et al., 2010). Therefore, high diversity of leaf litter in mixtures is expected to be an important determinant of the diversity and structure of decomposer communities and, consequently, litter decomposition (Gessner et al., 2010; Hättenschwiler et al., 2005; Trogisch et al., 2016).

Tropical montane rainforest ecosystems harbor an exceptional diversity of plant species (Beck & Ritcher, 2008; Homeier et al., 2008; Myers et al., 2000) and are associated with high numbers of animal species above‐ and belowground (Brehm et al., 2008; Maraun et al., 2008; Paulsch & Müller‐Hohenstein, 2008). However, the effect of plant litter diversity on decomposer communities and decomposition of litter in this ecosystems is little studied (Illig et al., 2008; Krashevska et al., 2017). Controlled experiments are needed to assess the effect of diversity and composition of litter species in mixtures on litter decomposition and microarthropod abundance.

Differences in leaf litter chemical composition are recognized as the main drivers of decomposition rates at the ecosystem level (Coûteaux et al., 1995; Hättenschwiler et al., 2005). Studies have reported positive, negative, but also no effects of litter mixtures on decomposition, with mixture effects typically related to variations in litter nutrient concentrations (Gartner & Cardon, 2004; Handa et al., 2014; Makkonen et al., 2012). However, differences in litter chemistry are not the only factors contributing to variations in litter decomposition in mixtures (Hättenschwiler, 2005; Hoorens et al., 2003). Physical leaf litter traits, such as toughness, surface structure, and shape, also contribute to microhabitat diversity and modify microenvironmental conditions of decomposer organisms, resulting in either accelerated or decelerated litter decomposition (Hansen & Coleman, 1998; Kaneko & Salamanca, 1999). Therefore, species identity, which encompasses chemical and physical characteristics, may well explain diversity effects on decomposition. Indeed, the effect of litter species identity has been found to be more powerful in explaining colonization of litter by invertebrates than litter diversity (Eissfeller et al., 2013; Korboulewsky et al., 2016; Schädler & Brandl, 2005; Vos et al., 2011; Wardle et al., 2006).

Commonly, studies investigating effects of litter diversity on litter decomposition focused on microorganisms and detritivore invertebrates (Gessner et al., 2010). Microorganisms are assumed to respond more sensitively to litter diversity than invertebrates as they directly depend on the variety of litter chemical compounds needed for metabolism and growth (Bardgett & Shine, 1999; Chapman et al., 2013). By contrast, the response of invertebrate detritivores, particularly the key decomposer groups Acari and Collembola, more strongly depends on the identity rather than diversity of leaf litter species and varies with the stage of litter decomposition (González & Seastedt, 2001; Illig et al., 2008; Kaneko & Salamanca, 1999; Korboulewsky et al., 2016; Wardle et al., 2006). Indeed, many decomposer microarthropods have the ability to select among co‐occurring leaf litter species according to litter palatability and/or the microorganisms colonizing the litter (Klironomos et al., 1992; Korboulewsky et al., 2016; Schneider & Maraun, 2005). Studies linking microbial‐dominated litter decomposition processes and colonization of litter by detritivore invertebrates are needed to uncover the mechanisms responsible for litter diversity effects on the structure and functioning of the decomposer system, particularly in tropical ecosystems characterized by high diversity of plant (tree) species.

In the present study, we investigated the effect of leaf litter diversity and identity on the colonization of litter by microorganisms and microarthropods including Acari and Collembola after 6 and 12 months of incubation in Ecuadorian montane rainforests. We hypothesized that (1) microbial growth and activity increase with litter diversity, but that the abundance of both Acari and Collembola relies more on litter identity. Additionally, assuming that microorganisms are limited by multiple nutrients (Demoling et al., 2007; Krashevska et al., 2010), we hypothesized that (2) nutrient availability increases and microbial stress conditions decrease with time and that (3) the presence of high‐quality litter benefits microorganisms. Further, assuming that Acari and Collembola prefer similar food resources and consume both leaf litter tissue and microorganisms (Dhooria, 2016; Ruess & Lussenhop, 2005; Seastedt, 1984), we hypothesized that (4) the abundance of Acari and Collembola increases as decomposition proceeds, particularly in presence of high‐quality litter.

## 2 MATERIALS AND METHODS

### 2.1 Study site

The study area is located in southern Ecuador on the eastern slopes of the Andean Cordillera. The site forms part of the Reserva Biológia San Francisco located on the northern borders of the Podocarpus National Park at 2,000 m a.s.l. (3°58′S, 79°04′W). The region is characterized by a semihumid climate with annual precipitation of about 2,200 mm and average annual temperature of 15.2°C (Bendix et al., 2006; Wullaert et al., 2009). The soil is Gley Cambisol with a soil pH of ~3.5 and a thick organic layer up to 35 cm comprised of mainly fermentation/humus material overlaid by litter material (Moser et al., 2007). The tropical rainforest is mostly undisturbed and holds an exceptionally high diversity of fauna and flora with *Rubiaceae, Melastomataceae,* and *Piperaceae* as dominant plant families (Beck & Ritcher, 2008; Brehm & Fiedler, 2005; Homeier et al., 2010; Maraun et al., 2008).

### 2.2 Experimental design

In September 2008, freshly fallen leaves of six common plant species at the study sites [*Cecropia andina* (Cuatrec.) (CA), *Dictyocaryum lamarckianum* (H. Wendl.) (DL), *Myrcia pubescens* (Humb. & Bonpl. ex Willd.) (MP), *Cavendishia zamorensis* (A. C. Sm.) (CZ), *Graffenrieda emarginata* (Ruiz & Pav.) (GE), and *Clusia* spp. (L.) (Cs); ordered by increasing C‐to‐N ratio, see Appendix 1] were collected, dried (60°C for 72 hr), and used to fill 20 × 20 cm and 4 mm nylon mesh litterbags. Initial chemical composition of the litter species is given in Appendix 1. The leaves used had no signs of herbivory, fungal infection or atypical texture or color. Large leaves exceeding the size of the litter bags were cut into ~5 × 5 cm pieces. Single‐species litterbags (12 g each) and mixtures with all possible two‐ (6 g per species) and four‐species combinations (3 g per species) were prepared, resulting in a total of 36 litterbag types with three levels of species diversity (1, 2, and 4 leaf litter species). Litterbags were randomly placed in the field on top of the undisturbed litter layer and fixed with nails in four blocks. Minimum distance between the blocks was 20 m. One replicate of each treatment was harvested after 6 and 12 months.

### 2.3 Analytical procedures

After harvest, material in each litterbag was separated into two subsamples of equal weight, disturbing the fauna as little as possible but ensuring that all litter types were present in both halves. One half was used for microarthropod extraction and the other for analysis of microbial parameters. Microarthropods were extracted by heat over one week using a modified high gradient extractor and then stored in 70% ethanol (Kempson et al., 1963; Macfadyen, 1961). Microarthropods were determined to group level [Collembola (Insecta), Oribatida, Mesostigmata, and Prostigmata (Acari)] using Schaefer (2018). The dry litter was sorted to species, weighed and used to measure litter chemical composition.

Microbial basal respiration (BR) and microbial biomass (*C*_mic_) were determined using an automated respirometer system (Scheu, 1992). BR (μl O_2_ g^−1^ dry weight hr^−1^) was measured at 22°C and calculated as mean of O_2_ consumption rates 10 to 20 hr after attachment of the samples to the respirometer system. *C*_mic_ was measured by the substrate‐induced respiration method (SIR; Anderson & Domsch, 1978; Beck et al., 1997). The maximum initial respiratory response (MIRR; µl O_2_ g^−1^ dry weight hr^−1^) was measured at 22°C after the addition of glucose to saturate the catabolic activity of microorganisms. MIRR was calculated as the average of the lowest three readings within the first 10 hr, and *C*_mic_ was calculated as *C*_mic_ = 38 × MIRR (mg/g dry weight). Respiration rates between the lowest (usually 3–6 hr after glucose addition) and highest reading were taken to calculate the slope of microbial growth (+*C*_Slope_). Data were ln‐transformed, and the slope determined by linear regression. The microbial metabolic quotient (*q*O_2_; μl O_2_ mg^−1^ *C*_mic_ hr^−1^) was calculated by dividing BR by *C*_mic_.

Leaf litter mass loss (*M*_loss_) was calculated as *M*_loss_ (%) = (*m*_0_ – *m*_1_/*m*_0_) × 100, where *m*_0_ is the initial dry weight and m_1_ the dry weight of leaf litter at harvest. To measure chemical composition, leaves from each of the six species were dried (65°C for 72 hr) and milled to particles <1 mm. Carbon (C) and nitrogen (N) were measured using a CN elemental analyzer (Vario EL III, Elementar). Total element analysis was measured by an ICP‐OES system (ICP‐OES, Optima 5300 DV, Perkin Elmer). Lignin and cellulose concentration were measured based on the methanol–chloroform–water (2:2:1) extraction method detailed in Allen et al. (1974). For litter mixtures, the proportion of elements per litterbag was calculated by proportionally summing the amount of the respective elements in the individual litter species. The chemical concentrations of elements, lignin and cellulose, were expressed as milligram per gram litter dry weight (dw).

### 2.4 Statistical analyses

Analyses were performed using R version 3.6.0 (R Core Team, 2014). Data were checked for normality and homoscedasticity using Shapiro–Wilk test and Bartlett's test (package “stats”). To improve normality and homoscedasticity, data were transformed using the “bestNormalize” function (package “CRAN”). Changes in *M*_loss_, *C*_mic_, BR, *q*O_2_, +*C*_Slope,_ and the abundance of microarthropod taxa (Collembola, Oribatida, Mesostigmata, and Prostigmata) were analyzed using individual linear mixed‐effects models (package “nlme”). In each model, the fixed factors litter diversity (LD; 1, 2, and 4 litter species), time of exposure (6 and 12 months), and the presence/absence all leaf litter species (litter identity; 1,0; CA, DL, MP, CZ, GE, and Cs), as well as the interactions (time × LD and time × litter identity), were fitted in a hierarchical design. Block was fitted first as random factor followed by the fixed factors litter diversity, time, interaction between litter diversity and time, and litter identity. To assess the relative importance of the six leaf litter species, analyses were repeated changing the order of fitting individual litter species and their interactions. *F*‐ and *p*‐values for individual litter species in the text and tables refer to those when fitted first (Schmid et al., 2002, 2017). Differences between means were inspected using Tukey's honestly significant difference test (package “emmeans”). Values presented in text are means ± *SD* of non−transformed data. Pearson correlation coefficients were calculated to investigate relationships between C‐to‐N ratio, *C*_mic_, *q*O_2_ and *M*_loss_, and the abundance of Collembola and Acari (package “stats”).

## 3 RESULTS

### 3.1 Initial litter chemistry

Initial N concentrations were highest in *C. andina*, followed by *D. lamarckianum*, *M. pubescens*, *C. zamorensis*, *G. emarginata,* and *Clusia* spp. (1.08%, 0.73%, 0.60%, 0.50%, 0.40%, and 0.40%, respectively), resulting in C‐to‐N ratios between 36.3 in *C. andina* and 107.2 in *Clusia* spp. (see Appendix 1 for details on litter chemistry). Lignin concentrations were generally high and varied between 63.9% in *Clusia* spp. to 42.6% in *G. emarginata*. By contrast, concentrations of cellulose were lowest in *Clusia* spp. (13.0%), low in *C. andina* (29.6%), but similar in the other four litter species varying between 35.8% and 40.7%. Concentrations of P and other litter elements also varied markedly between leaf litter species with P, Ca, Mg, K, and Fe being highest in *C. andina*, and P and Ca being lowest in *G. emarginata*.

### 3.2 Mass loss

**Figure 1:**
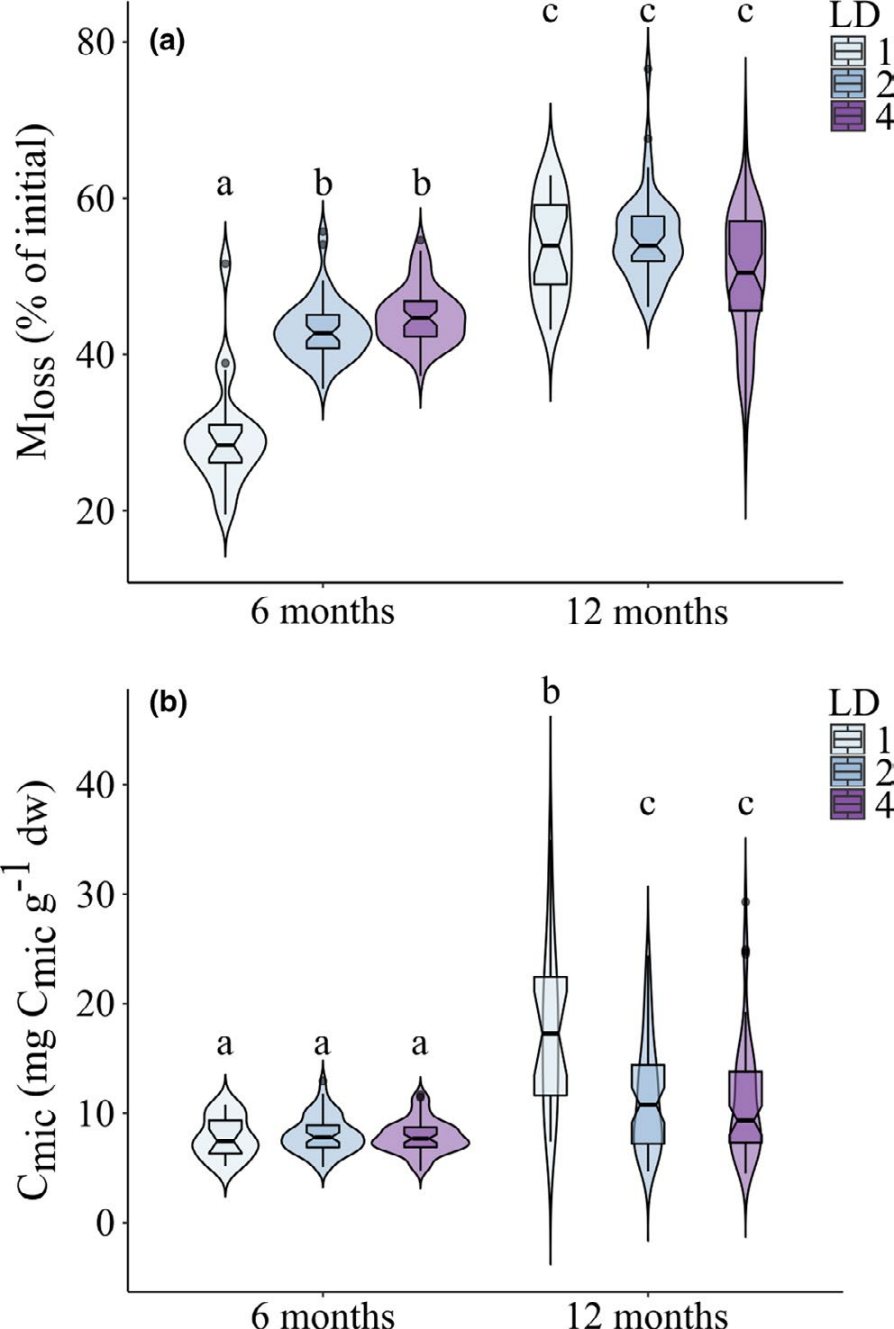
Effect of litter species diversity (LD; 1, 2, and 4 species) on (a) litter mass loss (*M*_loss_) and (b) litter microbial biomass (*C*_mic_) after 6 and 12 months of incubation in the field. Boxplots show medians and quantiles for each LD level. Violin plots illustrate kernel probability density. Different letters indicate significant differences (Tukey's HSD test, *p* < .05)

Generally, *M*_loss_ was higher after 12 than after 6 months of incubation with averages of 52.6% ± 7.1% and 41.8% ± 6.9% of initial, respectively (Table 1). *M*_loss_ varied significantly with species diversity but the effect depended on time (Table 1; Figure 1a); after 6 months *M*_loss_ was lower in single species (average of 29.6% ± 6.9%) compared to the two and four litter species treatments (43.1% ± 3.8 and 44.9% ± 3.6%, respectively), while after 12 months decomposition was similar in each of the litter diversity treatments. Further, *M*_loss_ varied significantly with litter species identity; however, this depended on time, with the effect generally being restricted to the first sampling date and to four of the six litter species (Table 1). At the first sampling date, *M*_loss_ increased in presence of *C. andina* from 39.7% ± 7.4% to 44.4% ± 5.1%, in presence of *C. zamorensis* from 40.5% ± 7.9% to 43.2% ± 5.3%*,* in presence of *G. emarginata* from 39.4% ± 7.6% to 44.8% ± 4.2%, and in presence of *Clusia* spp. from 39.6% ± 7.3% to 44.6% ± 5.1%. *M*_loss_ positively correlated with *C*_mic_, BR, *q*O_2_, +*C*_Slope,_ and the abundance of Collembola and Oribatida, but negatively with the litter C‐to‐N ratio (Pearson correlation coefficients; Table 2).

**Table 1:** *F*‐values of linear mixed‐effects models on the effect of litter species diversity (LD), time of exposure (Time), and leaf litter species identity [*Cecropia andina* (CA), *Dictyocaryum lamarckianum* (DL), *Myrcia pubescens* (MP), *Cavendishia zamorensis* (CZ), *Graffenrieda emarginata* (GE), *and Clusia* spp. (Cs)] on mass loss (*M*_loss_), microbial biomass (*C*_mic_), basal respiration (BR), microbial metabolic quotient (*q*O_2_), and the slopes of microbial growth after C addition (+*C*_Slope_)

	*df*	*M*_loss_	*C*_mic_	BR	*q*O_2_	+*C*_Slope_
LD	2, 239	26.32***	3.01*	1.12	2.01	2.03
Time	1, 239	244.03***	31.48***	78.10***	21.15***	24.61***
CA	1, 239	0.51	1.63	1.04	7.76**	1.21
DL	1, 239	1.09	<0.01	1.78	1.93	4.59*
MP	1, 239	2.09	<0.01	3.91*	0.46	0.70
CZ	1, 239	0.02	0.53	<0.01	4.49*	4.33*
GE	1, 239	0.43	0.11	0.04	<0.01	0.05
Cs	1, 239	0.97	0.05	0.02	<0.01	0.01
Time × LD	2, 239	43.44***	4.37**	1.43	1.27	1.73
Time × CA	1, 239	23.01***	0.12	0.01	<0.01	2.30
Time × DL	1, 239	0.91	0.47	0.11	0.66	3.89*
Time × MP	1, 239	1.76	0.60	3.13	0.60	0.59
Time × CZ	1, 239	7.25**	0.71	0.80	3.76*	2.48
Time × GE	1, 239	35.12***	6.76**	2.29	0.60	<0.01
Time × Cs	1, 239	21.73***	1.77	0.07	0.02	2.72

Note

*F*‐values represent those where the respective factor was fitted first. Significant effects are given in bold (*p < .05; **p < .01; ***p < .001).

Abbreviation: *df*, degrees of freedom.

**Table 2:** Pearson correlation coefficients between mass loss (*M*_loss_), microbial biomass (*C*_mic_), basal respiration (BR), microbial growth after C addition (+*C*_slope_), metabolic quotient (*q*O_2_), the abundance of Collembola, Oribatida, Mesostigmata, and Prostigmata, and litter C‐to‐N ratio

	*M*_loss_	*C*_mic_	BR	*q*CO_2_	+*C*_Slope_	Collembola	Oribatida	Mesostigmata	Prostigmata
*M*_loss_	1	—	—	—	—	—	—	—	—
*C*_mic_	0.30***	1	—	—	—	—	—	—	—
BR	0.42***	0.53***	1	—	—	—	—	—	—
*q*O_2_	0.20**	−0.16 ***	0.50***	1	—	—	—	—	—
+*C*_slope_	0.20**	0.23***	0.38***	0.07	1	—	—	—	—
Collembola	0.16**	0.09	0.04	−0.10	0.12	1	—	—	—
Oribatida	0.25***	0.08	0.13*	0.04	0.12	0.50***	1	—	—
Mesostigmata	−0.05	−0.07	−0.15*	−0.16*	−0.05	0.40***	0.40***	1	**—**
Prostigmata	0.05	0.02	<0.01	−0.11	0.07	0.37***	0.39***	0.48***	1
C‐to‐N	−0.24***	−0.16*	−0.19**	0.05	−0.15*	−0.15*	−0.01	−0.07	−0.19***

Note

Significant correlations are given in bold (*p < .05; **p < .01; ***p < .001).

### 3.3 Microbial parameters

Parallel to *M*_loss_, the microbial parameters *C*_mic_, BR, *q*O_2,_ and +*C*_Slope_ significantly increased from 6 to 12 months (Table 1; for means see Appendix 2). Among microbial parameters, only *C*_mic_ varied with litter diversity. Unlike *M*_loss_, the effect of litter diversity was restricted to the second sampling date, decreasing in the order one > two > four litter species (Figure 1b). Further, *C*_mic_ also varied with litter species identity, but the effect was restricted to treatments with *G. emarginata* and depended on time. At the second sampling date, *C*_mic_ decreased from 15.23 ± 11.74 to 11.58 ± 7.37 mg *C*_mic_ g^−1^ dw in litterbags without and with *G. emarginata*, respectively. The other microbial parameters only were significantly affected by litter species identity, with the effects in part varying with time (Table 1). BR decreased significantly in presence of *M. pubescens* from an average of 157.3 ± 107.7 to 133.1 ± 69.40 μl O_2_ mg^−1^ *C*_mic_ hr^−1^ in litterbags without and with *M. pubescens*, respectively. *q*O_2_ decreased from 14.90 ± 5.65 to 13.50 ± 4.18 μl O_2_ mg^‐1^ *C*_mic_ hr^−1^ in presence of *C. andina*, irrespective of sampling date, but it increased from 14.44 ± 5.37 to 16.91 ± 7.45 45 μl O_2_ mg^‐1^ *C*_mic_ hr^−1^ in presence of *C. zamorensis* at the second sampling date. +*C*_Slope_ decreased significantly from 0.0097 ± 0.0149 to 0.0061 ± 0.0131 in presence of *C. zamorensis* irrespective of sampling date, but in presence of *D. lamarckianum* it increased from 0.0086 ± 0.0195 to 0.0151 ± 0.0180 after the second sampling.

Pearson correlation coefficients indicated that *C*_mic_ positively correlated with *M*_loss_, BR and +*C*_Slope_, but negatively with *q*O_2_ and the litter C‐to‐N ratio. BR positively correlated with *M*_loss_, *C*_mic,_ *q*O_2_, +*C*_Slope,_ and the abundance of Oribatida, but negatively with the abundance of Mesostigmata and the litter C‐to‐N ratio. *q*O_2_ positively correlated with *M*_loss_ and BR, but negatively with *C*_mic_ and the abundance of Mesostigmata. +*C*_Slope_ positively correlated with *M*_loss_, *C*_mic_, and BR, but negatively with the litter C‐to‐N ratio (Table 2).

### 3.4 Microarthropods

**Figure 2:**
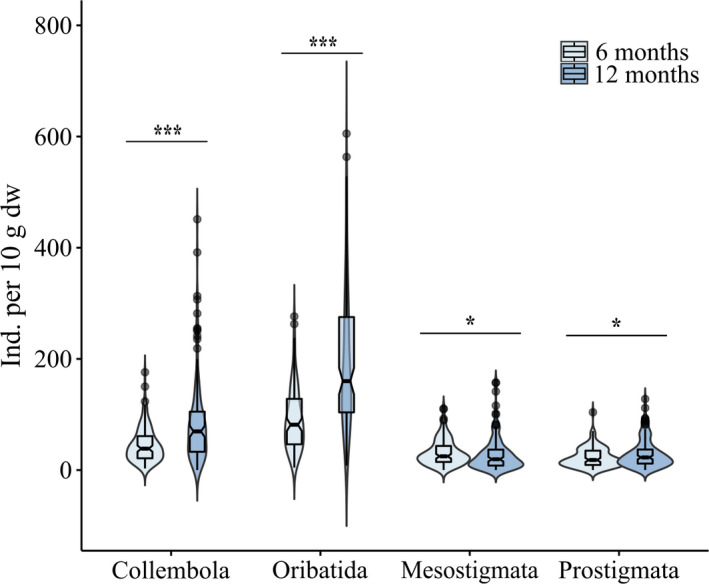
Abundance of Collembola, Oribatida, Mesostigmata, and Prostigmata in litterbags after 6 and 12 months of incubation in the field**.** Boxplots show medians and quantiles for each date of exposure. Violin plots illustrate kernel probability density. ***p < .001; *p < .05

The number of Collembola, Oribatida, and Prostigmata significantly increased from 6 to 12 months, but the abundance of Mesostigmata decreased (Table 3; Figure 2; for means, see Appendix 3). None of the soil microarthropod taxa investigated varied with litter diversity, although they did vary significantly with litter species identity (Table 3). Collembola abundance (25.3% of total microarthropods; overall mean of 70 ± 80 ind. 10 g^−1^ litter dw) increased significantly in presence of *C. andina* by 43.4% and in presence of *G. emarginata* by 29.2%, but decreased in presence of *D. lamarckianum* and *C. zamorensis* by 39.1% and 38.1%, respectively (Appendices 3 and 4). However, the effect varied with time for *D. lamarckianum* and *C. zamorensis* (Table 3); in the presence of these species, the reduction was most pronounced after 12 months (from 60 ± 42 to 123 ± 132 and from 62 ± 38 to 124 ± 135 ind. 10 g^−1^ litter dw, respectively). The abundance of Oribatida (53.7% of total microarthropods; overall mean 146 ± 119 ind. 10 g^−1^ litter dw) increased significantly in litterbags containing *G. emarginata* or *Clusia* spp. from 133 ± 119 to 162 ± 118 and from 131 ± 99 to 163 ± 138 ind. 10 g^−1^ litter dw, respectively. Further, Mesostigmata abundance (11.1% of total microarthropods; overall mean of 30 ± 27 ind. 10 g^−1^ litter dw) decreased significantly by 24.5% from 34 ± 31 to 26 ± 21 ind. 10 g^−1^ litter dw in the presence of *C. zamorensis*. Prostigmata abundance (9.5% of total microarthropods; overall mean of 26 ± 22 ind. 10 g^−1^ litter dw) increased significantly in litterbags where *C. andina* or *Clusia* spp. were present. With the former, it increased by 28.1% from 23 ± 22 to 29 ± 22 ind. 10 g^−1^ litter dw, while in the presence of the latter the effect was restricted to the second sampling date, increasing by 23.1% from 27 ± 25 to 33 ± 26 ind. 10 g^−1^ litter dw.

**Table 3:** *F*‐values of linear mixed‐effects models on the effect of litter species diversity (LD), time of exposure (Time), and leaf litter species identity [*Cecropia andina* (CA), *Dictyocaryum lamarckianum* (DL), *Myrcia pubescens* (MP), *Cavendishia zamorensis* (CZ), *Graffenrieda emarginata* (GE), *and Clusia* spp. (Cs)] on the abundance of Collembola, Oribatida, Mesostigmata, and Prostigmata

	*df*	Collembola	Oribatida	Mesostigmata	Prostigmata
LD	2, 239	0.15	1.41	0.75	0.74
Time	1, 239	28.08***	78.95***	4.93*	4.22*
CA	1, 239	15.83***	1.50	2.86	7.92**
DL	1, 239	13.34***	0.34	0.05	0.66
MP	1, 239	<0.01	0.85	0.37	2.74
CZ	1, 239	8.80**	2.73	4.61*	2.06
GE	1, 239	7.59**	5.98**	2.43	1.56
Cs	1, 239	<0.01	4.24*	0.07	0.02
Time × LD	2, 239	2.80	0.61	0.71	0.39
Time × CA	1, 239	0.14	0.59	2.26	3.08
Time × DL	1, 239	8.04**	0.02	1.01	0.42
Time × MP	1, 239	0.85	0.30	0.23	0.03
Time × CZ	1, 239	4.52*	0.01	0.01	<0.01
Time × GE	1, 239	0.22	0.03	0.14	0.33
Time × Cs	1, 239	0.44	0.02	0.04	4.25*

Note

*F*‐values represent those where the respective factor was fitted first. Significant effects are given in bold (*p < .05; **p < .01; ***p < .001).

Abbreviation: *df*, degrees of freedom.

Pearson correlation coefficients indicated that Collembola abundance positively correlated with *M*_loss_ and the abundance of Oribatida, Mesostigmata, and Prostigmata, but negatively with the litter C‐to‐N ratio. Oribatida abundance positively correlated with *M*_loss_, BR, and the abundance of Collembola, Mesostigmata, and Prostigmata. Mesostigmata abundance positively correlated with the abundance of Collembola, Oribatida, and Prostigmata, but negatively with BR and *q*O_2_. Prostigmata abundance positively correlated with the abundance of Collembola, Oribatida, and Mesostigmata, but negatively with litter C‐to‐N ratio (Table 2).

## 4 DISCUSSION

### 4.1 Litter diversity

Contrary to our first hypothesis, *C*_mic_ decreased rather than increased with increasing litter diversity after one year of exposure in the field (Figure 1). Leaves of tropical forest trees are of low nutritional quality and contain high concentrations of structural compounds and secondary metabolites, typically higher than those in trees of temperate forests (Cárdenas et al., 2015; Coley & Barone, 1996; Hallam & Read, 2006). Secondary metabolites, particularly polyphenols known to suppress microorganisms by inhibiting enzyme activity (Hättenschwiler & Vitousek, 2000; Hoorens et al., 2003), are important drivers of decomposition processes particularly in tropical rainforests (Coq et al., 2010). Potentially, secondary compounds, such as polyphenols, detrimentally affected litter microorganisms in a systemic way resulting in a decrease in *C*_mic_, thereby resulting in a negative complementarity effect in leaf litter mixtures (Chomel et al., 2016; Ristok et al., 2019). The fact that BR, *q*O_2,_ and +*C*_Slope_ were not significantly affected by litter diversity suggests that higher leaf litter diversity does not necessarily result in an increase in the availability of nutrient and carbon resources in this tropical rainforest. Rather, the results suggest that litter diversity increases the exposure of microorganisms to secondary leaf litter compounds, detrimentally affecting their activity. Due to the preferential decay of labile litter compounds, the concentration of secondary compounds as well as recalcitrant structural compounds, such as lignin, may increase during litter decomposition, thereby reducing litter decomposition at later stages of litter decay, as has previously been suggested for litter at our study sites (Butenschoen et al., 2014; Marian et al., 2017).

Similar to *C*_mic_, *M*_loss_ significantly increased in single litter species treatments after one year of exposure underscoring the correlation between (Table 2). Changes in the chemical composition of litter material throughout the decomposition process alter the structure and functioning of microbial communities and thus affect the rate at which litter material is decomposed (Berg & McClaugherty, 2008). Notably, *M*_loss_ increased with litter diversity after 6 months of exposure; however, the effect was no longer present after 12 months. Presumably, this reflects reliance of the early microbial community on labile litter compounds, which were more abundant in leaf litter mixtures (Pérez Harguindeguy et al., 2008; Rinkes et al., 2014). However, as decomposition proceeded, the remaining more recalcitrant compounds accumulated and their decomposition was independent of litter diversity.

In contrast with *C*_mic_ and *M*_loss_, the abundance of microarthropods was not affected by litter diversity (Table 3). Some previous studies found mixtures to promote the abundance of microarthropods (Hansen, 2000; Hättenschwiler & Gasser, 2005; Migge et al., 1998; Schädler & Brandl, 2005), while others did not find evidence that litter diversity beneficially affects microarthropods (Bluhm et al., 2019; Ilieva‐Makulec et al., 2006; Korboulewsky et al., 2016; Patoine et al., 2020; Scheu et al., 2003). Our results agree with the latter findings and support the results of Marian et al. (2018) suggesting that litter diversity in this tropical rainforest neither improves habitat conditions nor the availability of resources for microarthropods, at least during early stages of decomposition. Indeed, detritivore microarthropods are considered to comprise predominantly generalist feeders colonizing a range of forest types and therefore are rather insensitive to changes caused by litter mixing (Ball et al., 2014; Gergócs & Hufnagel, 2016; Patoine et al., 2020; Wardle et al., 2006). However, even though litter diversity did not affect microarthropod abundance, it may still have fostered the diversity of microarthropods, as has been shown for other soil organisms, such as testate amoebae at our study site (Krashevska et al., 2017).

### 4.2 Exposure time

Generally, *M*_loss_ increased with time parallel to microbial parameters. Litter decomposition at our study site can be divided into three phases, with the early phase lasting for about 12 months (Marian et al., 2017). This early phase of decomposition is characterized by the loss of labile C compounds via leaching and by the growth of opportunistic microorganisms that form new soluble compounds (Berg & McClaugherty, 2008), and this likely explains the close link between *M*_loss_ and microbial activity and growth (Table 2). However, contrary to our second hypothesis, the increase in *q*O_2_ values between 6 and 12 months of exposure indicates that microorganisms increasingly suffered from stress conditions later during exposure. Stress conditions result in less efficient use of C compounds and increased investment into maintenance metabolism (Ndaw et al., 2009; Yan et al., 2003). Presumably, toward the end of the early litter decomposition stage microorganisms increasingly competed for resources as easily decomposable leaf litter compounds vanished (Fontaine et al., 2003; Poll et al., 2008; Rinkes et al., 2011). The parallel increase in the +*C*_Slope_ with time suggests that this was associated with less efficient nutrient capture by microorganisms pointing toward a switch from predominant limitation by nutrients early during exposure to the limitation by easily available carbon resources later (Laganière et al., 2010; Sall et al., 2003). Early stages of litter decay in the studied tropical montane rainforest might be associated with high abundance of mycorrhizal fungi (Marian et al., 2017). The C input that mycorrhizal fungi obtain from plants may allow them to efficiently compete with saprotrophic fungi for nutrients, even though their enzymatic capability is typically inferior to that of saprotrophic fungi (Camenzind & Rillig, 2013; Hodge et al., 2001). Indeed, the assumption that mycorrhizal and saprotrophic fungi interact antagonistically early during litter decomposition at our study site is supported by earlier studies (Marian et al., 2019; Sánchez‐Galindo et al., 2019).

Parallel to microbial parameters, the abundance of all microarthropod taxa studied increased with time, with the exception of Mesostigmata. Mesostigmata commonly hunt in the litter for other microarthropods, particularly Collembola, Astigmata and weakly sclerotized Oribatida (Koehler, 1997; Schneider & Maraun, 2009). Although variations in the abundance of Mesostigmata were closely linked to the abundance of Collembola and Oribatida (Table 2), the fact that their abundance decreased with time likely reflects that Mesostigmata in the litterbags were not only feeding on microarthropods, but also on other organisms, presumably Nematoda, insect larvae and eggs. Indeed, some species of Mesostigmata may preferentially colonize certain microhabitats to hunt for prey such as Nematoda (Heidemann et al., 2014; Klarner et al., 2013).

The increase in the abundance of the microarthropod decomposers Collembola and Oribatida with time indicates that changes during the initial stages of decomposition influence both groups in a similar way. Surprisingly, Collembola and Oribatida abundance was not closely associated with microbial biomass (Table 2) even though microorganisms are their major food resource (Dhooria, 2016; Maraun et al., 2003; Scheu et al., 2005). Rather, the stage of litter decomposition within the early decomposition phase (i.e., 6 vs. 12 months) appears to be the more important driver of the abundance of microarthropod decomposers. Indeed, litter material that is highly colonized by microorganisms becomes more palatable for microarthropods (Bardgett, 2005; Das & Joy, 2009), which at least in part is due to the reduction in plant secondary compounds such as phenols (Asplund et al., 2013; Coulis et al., 2009). Overall, our results support earlier findings at this study site in that the role of litter resources for the nutrition of decomposer microarthropods increases with litter decomposition (Marian et al., 2018). Moreover, the parallel increase in the abundance of Prostigmata suggests that the increase in the abundance of decomposer microarthropod prey benefitted higher trophic levels.

### 4.3 Leaf litter identity

The presence of specific plant leaf litter species in mixtures might increase or decrease the rate at which the litter decomposes (Hector et al., 2000; Hoorens et al., 2003, 2010). Variation can be attributed predominantly to differences in litter quality among the component species in mixtures (Gartner & Cardon, 2004; Hättenschwiler et al., 2005). Indeed, litter decomposition and colonization of the litter by microarthropods in our study were related to the initial chemical composition of the litter species. Our third hypothesis was supported by the beneficial effects of high‐quality *C. andina* litter. Presence of this litter species significantly decreased *q*O_2_ values and increased the abundance of Collembola and Prostigmata. *C. andina* had high initial N and P concentrations, and low lignin content (see Appendix 1), providing readily available nutrients, reducing nutrient stress for microorganisms, and thereby contributing to an increase in *C*_mic_. Increased microbial C use efficiency may also have resulted from a shift in microbial community composition toward high‐energy‐efficient species (Dilly & Munch, 1996), for example, from opportunistic bacteria to fungi able to break down complex litter compounds (Chapman et al., 2013). Changes in microbial community composition probably were driven by increasing concentrations of recalcitrant litter compounds favoring saprotrophic fungi able to degrade these compounds, which in turn beneficially affected decomposers, such as Collembola and Oribatida, feeding on these fungi and the litter materials degraded by them.

The high *q*O_2_ and the +*C*_Slope_ values after 12 months of exposure reflected the low quality of *D. lamarckianum*, *C. zamorensis,* and *G. emarginata* litter, and presumable scarcity of easily accessible C resources to microorganisms. All these litter species were characterized by low initial N and P concentrations, and high concentrations of lignin and cellulose (Appendix 1). The concentrations of lignin and cellulose serve as indicator of litter quality and as predictor of litter decomposition (Berg, 2014; Fioretto et al., 2005). Cellulose not entrapped in lignin degrades rapidly during early stages of decomposition, and this contributes to the release of N and P, typical elements limiting microbial growth (Berg, 2014; Berg & McClaugherty, 2008; Hobbie et al., 2012). However, during this stage, labile compounds are commonly used by opportunistic microorganisms (Cornelissen et al., 1999; Fioretto et al., 2005), impeding the growth of microorganism able to degrade recalcitrant litter compounds (Ilieva‐Makulec et al., 2006). Therefore, by the end of the early stage of litter decomposition, structural compounds become relatively more abundant and reduce resource quality, which differentially affects microorganisms and microarthropods, as indicated by the lower abundance of Collembola in litter of *C. zamorensis* and *D. lamarckianum*. Interestingly, the decrease in *C*_mic_ after 12 months in litterbags containing *G. emarginata* was associated with high abundance of decomposer microarthropods, suggesting that there is no close relationship between decomposer microarthropods and bulk microbial biomass in litter. This conclusion is also supported by the lack of significant correlations between *C*_mic_ and decomposer microarthropod abundances (Table 2).

The correlation between the abundance of Collembola and Oribatida and litter *M*_loss_ presumably reflects that these microarthropods benefited from both higher quality litter and by microorganisms colonizing the litter at later stages of decay. The significant negative correlation between Collembola abundance and litter C‐to‐N ratio (Table 2) indicates that Collembola heavily rely on litter quality. However, contrary to our fourth hypothesis, the differential responses of microarthropods to litter species suggest that leaf litter chemical composition alone is insufficient to explain variations in the abundance of soil microarthropods, as has been suggested in earlier studies (González & Seastedt, 2001; Hoorens et al., 2010; Kaneko & Salamanca, 1999). This is most strongly supported by the greater abundance of Oribatida in litterbags containing *Clusia* spp. litter, which was of particular low quality. This indicates that physical litter characteristics such as toughness and structure might play a more important role in driving soil microarthropod abundance than litter chemistry and the degree of microbial colonization.

## 5 CONCLUSIONS

The results of our study showed that higher levels of litter diversity may negatively affect soil microbial biomass and mass loss in the studied tropical montane rainforest, presumably due to the accumulation of recalcitrant compounds and the generally low quality of the leaf litter material. Notably, the response of microbial parameters and microarthropod abundance to litter identity was more pronounced than to litter diversity, with the differential responses of soil biota to litter identity in part being due to differences in the initial chemical composition of litter species. Generally, the results indicate that both microarthropods and microorganisms benefit from larger amounts of easily available litter resources during early stages of decomposition, highlighting the importance of litter quality as driver of the abundance and activity of decomposer organisms. However, the results also indicate that litter traits, related to the physical structure of litter, may be more important to decomposer invertebrates than litter chemistry and gross microbial characteristics of litter such as microbial biomass. Overall, our findings indicate that litter species identity functions as major driver of the abundance and activity of soil organisms, and thereby exert distinct effects on ecosystem processes such as decomposition and nutrient mobilization.

## CONFLICT OF INTEREST

None declared.

## AUTHOR CONTRIBUTIONS

**Laura Sanchez Galindo:** Data curation (equal); Formal analysis (lead); Investigation (equal); Validation (equal); Visualization (lead); Writing‐original draft (lead). **Dorothee Sandmann:** Data curation (equal); Formal analysis (equal); Investigation (equal); Methodology (supporting); Writing‐review & editing (equal). **Franca Marian:** Data curation (equal); Formal analysis (equal); Investigation (equal); Writing‐review & editing (equal). **Valentyna Krashevska:** Data curation (equal); Investigation (equal); Writing‐review & editing (equal). **Mark Maraun:** Conceptualization (equal); Funding acquisition (equal); Investigation (equal); Methodology (equal); Writing‐review & editing (equal). **Stefan Scheu:** Conceptualization (equal); Formal analysis (equal); Funding acquisition (equal); Investigation (equal); Methodology (equal); Project administration (lead); Resources (lead); Supervision (equal); Validation (equal); Writing‐original draft (equal).

## Supporting information

Supplementary Material

## Data Availability

All data are available as electronic supplementary material.

## Appendix 1

Initial chemical composition of the litter species used in the experiment. The analyses were performed in triplicate using bulk samples. Data are given in percentages of dry mass; nd = not detected.

**Table:**

	*Cecropia andina*	*Dictyocaryum lamarckianum*	*Myrcia pubescens*	*Cavendishia zamorensis*	*Graffenrieda emarginata*	*Clusia* spp.
C	39.30 ± 3.65	41.25 ± 1.70	39.61 ± 3.65	41.74 ± 0.01	40.28 ± 1.68	42.80 ± 3.65
N	1.08 ± 0.02	0.73 ± 0.01	0.60 ± 0.02	0.50 ± 0.01	0.40 ± 0.16	0.40 ± 0.02
C‐to‐N	36.29 ± 2.91	58.59 ± 1.25	65.64 ± 4.62	84.64 ± 0.01	91.29 ± 1.68	107.21 ± 6.00
Lignin	46.67 ± 6.37	52.40 ± 9.83	50.53 ± 9.07	51.73 ± 13.95	42.60 ± 8.40	63.93 ± 10.10
Cellulose	29.60 ± 6.28	40.73 ± 4.29	35.80 ± 7.27	39.53 ± 3.33	40.40 ± 6.73	13.00 ± 3.37
Al	1.88 ± 0.65	0.14 ± 0.08	0.18 ± 0.06	0.23 ± 0.02	2.41 ± 0.33	0.13 ± 0.01
Ca	17.32 ± 0.82	1.13 ± 0.08	1.07 ± 0.02	6.11 ± 0.83	1.07 ± 0.02	3.07 ± 0.82
Fe	2.03 ± 0.05	1.18 ± 0.02	0.29 ± 0.08	0.09 ± 0.03	0.30 ± 0.03	0.06 ± 0.02
K	3.05 ± 0.01	0.37 ± 0.09	1.23 ± 0.09	1.08 ± 0.01	1.08 ± 0.03	1.65 ± 0.09
Mg	3.22 ± 0.73	1.25 ± 0.09	1.22 ± 0.09	1.72 ± 0.09	1.72 ± 0.09	1.55 ± 0.09
Mn	0.11 ± 0.01	0.31 ± 0.03	0.14 ± 0.09	0.06 ± 0.01	0.26 ± 0.09	0.48 ± 0.09
Na	nd	0.03 ± 0.02	0.30 ± 0.02	nd	nd	nd
P	0.48 ± 0.08	0.21 ± 0.08	0.22 ± 0.10	0.27 ± 0.10	0.12 ± 0.08	0.25 ± 0.08

## Appendix 2

Means of microbial parameters (*C*_mic_, microbial biomass carbon; BR, basal respiration, *q*O_2_, microbial specific respiration; +*C*_Slope_, the slopes of microbial growth after C addition). LD, litter diversity (LD1, one species; LD2, two species; LD4, four species); CA, *Cecropia andina*; DL, *Dictyocaryum lamarckianum*; MP, *Myrcia pubescens*; CZ, *Cavendishia zamorensis*; GE, *Graffenrieda emarginata*; Cs, *Clusia* spp. Values are means ± *SD*.

**Table:**

			*C*_mic_ (mg *C*_mic_ g^−1^ dw)	BR (μl O_2_ mg^−1^ *C*_mic_ hr^−1^)	*q*O_2_ (μl O_2_ mg^−1^ *C*_mic_ hr^−1^)	+*C*_Slope_
LD	1		13.30 ± 8.99	154.57 ± 97.62	12.97 ± 5.55	0.0113 ± 0.0208
	2		11.01 ± 9.74	152.42 ± 105.28	14.78 ± 5.71	0.0066 ± 0.0111
	4		10.10 ± 6.12	138.35 ± 79.27	14.27 ± 4.22	0.0084 ± 0.0121
Time	6 months		8.28 ± 4.19	101.7 ± 19.5	12.90 ± 2.21	0.0042 ± 0.0035
	12 months		13.62 ± 10.18	191.1 ± 114.0	15.65 ± 6.56	0.0117 ± 0.0190
CA	Presence		11.12 ± 6.22	148.50 ± 94.82	13.50 ± 4.18	0.0089 ± 0.0157
	Absence		10.81 ± 9.56	144.73 ± 91.97	14.90 ± 5.65	0.0075 ± 0.0114
DL	Presence		10.58 ± 6.98	150.09 ± 89.94	14.74 ± 4.06	0.0097 ± 0.0140
	Absence		11.28 ± 9.20	143.14 ± 96.06	13.85 ± 5.82	0.0067 ± 0.0130
MP	Presence		10.53 ± 9.53	133.07 ± 69.40	14.15 ± 4.14	0.0074 ± 0.0104
	Absence		11.29 ± 6.97	157.33 ± 107.72	14.37 ± 5.75	0.0088 ± 0.0156
CZ	Presence		10.33 ± 8.53	143.71 ± 89.58	14.94 ± 5.75	0.0065 ± 0.0117
	Absence		11.54 ± 7.89	149.02 ± 96.61	13.63 ± 4.27	0.0097 ± 0.0149
GE	Presence		10.31 ± 6.80	141.93 ± 94.72	14.34 ± 5.10	0.0079 ± 0.0121
	Absence		11.46 ± 9.17	149.98 ± 91.98	14.21 ± 5.08	0.0084 ± 0.0146
Cs	Presence		10.36 ± 6.53	142.19 ± 90.71	14.32 ± 5.05	0.0080 ± 0.0101
	Absence		11.42 ± 9.33	149.78 ± 95.13	14.24 ± 5.12	0.0083 ± 0.0157
Time × LD						
LD1	6 months		7.79 ± 1.85	96.07 ± 18.97	12.65 ± 2.50	0.0045 ± 0.0031
	12 months		18.81 ± 9.92	213.06 ± 109.53	13.29 ± 7.53	0.0182 ± 0.0280
LD 2	6 months		8.03 ± 1.71	103.05 ± 19.08	13.03 ± 1.91	0.0041 ± 0.0036
	12 months		13.97 ± 13.06	201.79 ± 130.59	16.53 ± 7.48	0.0091 ± 0.0149
LD 4	6 months		8.67 ± 5.94	102.47 ± 20.01	12.86 ± 2.38	0.0043 ± 0.0036
	12 months		11.52 ± 6.01	174.23 ± 98.28	15.69 ± 5.11	0.0126 ± 0.0158
Time × Litter identity						
CA	Presence	6 months	8.64 ± 1.66	103.19 ± 19.44	12.12 ± 2.00	0.0037 ± 0.0044
	Absence	6 months	7.99 ± 5.44	100.50 ± 19.58	13.53 ± 2.20	0.0047 ± 0.0026
	Presence	12 months	13.61 ± 7.91	193.81 ± 116.56	14.89 ± 5.23	0.0142 ± 0.0206
	Absence	12 months	13.63 ± 11.76	188.97 ± 112.63	16.27 ± 7.46	0.0104 ± 0.0155
DL	Presence	6 months	8.25 ± 5.81	103.55 ± 19.70	13.62 ± 2.31	0.0044 ± 0.0034
	Absence	6 months	8.31 ± 1.79	100.05 ± 19.29	12.24 ± 1.92	0.0041 ± 0.0037
	Presence	12 months	12.90 ± 7.31	196.63 ± 107.35	15.86 ± 5.04	0.0151 ± 0.0180
	Absence	12 months	14.29 ± 12.21	186.23 ± 120.22	15.45 ± 7.71	0.0094 ± 0.017
MP	Presence	6 months	8.27 ± 5.94	98.27 ± 16.49	13.03 ± 2.36	0.0041 ± 0.0033
	Absence	6 months	8.29 ± 1.74	104.52 ± 21.33	12.79 ± 2.10	0.0043 ± 0.0038
	Presence	12 months	12.80 ± 11.71	167.88 ± 83.56	15.27 ± 5.14	0.0106 ± 0.0136
	Absence	12 months	14.30 ± 8.76	210.15 ± 131.38	15.96 ± 7.56	0.0133 ± 0.0209
CZ	Presence	6 months	8.07 ± 1.52	103.63 ± 18.99	12.98 ± 1.81	0.0039 ± 0.0028
	Absence	6 months	8.48 ± 5.68	99.88 ± 19.92	12.82 ± 2.56	0.0046 ± 0.0041
	Presence	12 months	12.59 ± 11.57	183.80 ± 112.03	16.91 ± 7.45	0.0092 ± 0.0160
	Absence	12 months	14.60 ± 8.61	198.16 ± 116.24	14.44 ± 5.38	0.0149 ± 0.0194
GE	Presence	6 months	9.03 ± 5.98	105.82 ± 20.97	12.71 ± 2.31	0.0039 ± 0.0031
	Absence	6 months	7.69 ± 1.62	98.45 ± 17.71	13.04 ± 2.14	0.0045 ± 0.0038
	Presence	12 months	11.58 ± 7.37	178.04 ± 122.51	15.98 ± 6.45	0.0119 ± 0.0159
	Absence	12 months	15.23 ± 11.74	201.52 ± 106.48	15.39 ± 6.68	0.0122 ± 0.0196
Cs	Presence	6 months	8.40 ± 6.07	99.02 ± 21.32	12.88 ± 2.48	0.0056 ± 0.0042
	Absence	12 months	8.18 ± 1.60	103.83 ± 17.76	12.91 ± 1.99	0.0032 ± 0.0026
	Presence	6 months	12.32 ± 6.44	185.36 ± 111.16	15.75 ± 6.41	0.0105 ± 0.0133
	Absence	12 months	14.65 ± 12.31	195.73 ± 116.75	15.57 ± 6.72	0.0133 ± 0.0210

## Appendix 3

Means of microarthropod abundance. Values are means ± *SD*. For legend, see Appendix 2.

**Table:**

			Collembola (ind. 10 g^−1^)	Oribatida (ind. 10 g^−1^)	Mesostigmata (ind. 10 g^−1^)	Prostigmata (ind. 10 g^−1^)
LD	1		73 ± 79	132 ± 116	26 ± 24	22 ± 18
	2		71 ± 67	150 ± 128	32 ± 29	26 ± 23
	4		67 ± 91	147 ± 113	30 ± 26	27 ± 23
Time	6 months		46 ± 31	90 ± 55	32 ± 23	22 ± 17
	12 months		93 ± 104	201 ± 137	29 ± 30	29 ± 26
CA	Presence		83 ± 99	141 ± 117	33 ± 27	29 ± 22
	Absence		58 ± 59	150 ± 121	28 ± 27	23 ± 22
DL	Presence		52 ± 37	141 ± 106	30 ± 27	25 ± 23
	Absence		85 ± 103	150 ± 130	30 ± 27	27 ± 22
MP	Presence		71 ± 98	144 ± 125	30 ± 28	24 ± 22
	Absence		69 ± 63	147 ± 114	30 ± 26	27 ± 22
CZ	Presence		53 ± 35	132 ± 95	26 ± 21	25 ± 23
	Absence		85 ± 105	159 ± 137	34 ± 31	27 ± 22
GE	Presence		80 ± 99	162 ± 118	34 ± 29	28 ± 24
	Absence		62 ± 61	133 ± 119	27 ± 25	24 ± 20
Cs	Presence		75 ± 104	163 ± 138	30 ± 28	27 ± 23
	Absence		65 ± 56	131 ± 99	30 ± 26	25 ± 22
Time × LD						
LD1	6 months		35 ± 24	76 ± 59	27 ± 23	18 ± 13
	12 months		111 ± 96	189 ± 132	25 ± 25	26 ± 22
LD 2	6 months		47 ± 35	93 ± 53	32 ± 26	21 ± 14
	12 months		94 ± 82	207 ± 154	32 ± 32	32 ± 28
LD 4	6 months		48 ± 29	93 ± 57	33 ± 21	25 ± 21
	12 months		87 ± 123	200 ± 128	27 ± 31	28 ± 25
Time × Litter identity						
CA	Presence	6 months	56 ± 34	88 ± 54	37 ± 26	28 ± 20
	Absence	6 months	37 ± 26	92 ± 57	27 ± 20	17 ± 15
	Presence	12 months	111 ± 131	193 ± 137	28 ± 28	30 ± 24
	Absence	12 months	78 ± 74	208 ± 140	29 ± 32	28 ± 27
DL	Presence	6 months	44 ± 30	90 ± 56	33 ± 25	23 ± 20
	Absence	6 months	47 ± 32	91 ± 55	30 ± 21	21 ± 15
	Presence	12 months	60 ± 42	192 ± 119	27 ± 28	26 ± 25
	Absence	12 months	123 ± 132	209 ± 154	30 ± 33	32 ± 27
MP	Presence	6 months	43 ± 27	87 ± 48	30 ± 20	22 ± 20
	Absence	6 months	48 ± 34	93 ± 61	33 ± 25	22 ± 15
	Presence	12 months	98 ± 131	201 ± 151	31 ± 35	26 ± 24
	Absence	12 months	89 ± 77	201 ± 129	27 ± 27	32 ± 27
CZ	Presence	6 months	44 ± 30	85 ± 54	28 ± 20	21 ± 16
	Absence	6 months	47 ± 32	96 ± 57	34 ± 26	23 ± 19
	Presence	12 months	62 ± 38	179 ± 103	23 ± 22	28 ± 27
	Absence	12 months	124 ± 135	222 ± 163	34 ± 37	30 ± 24
GE	Presence	6 months	50 ± 34	102 ± 61	35 ± 22	25 ± 20
	Absence	6 months	42 ± 29	81 ± 49	29 ± 23	20 ± 15
	Presence	12 months	109 ± 131	221 ± 130	33 ± 35	32 ± 28
	Absence	12 months	81 ± 76	185 ± 144	25 ± 26	27 ± 24
Cs	Presence	6 months	45 ± 27	100 ± 57	30 ± 19	21 ± 17
	Absence	6 months	46 ± 34	82 ± 53	33 ± 26	23 ± 18
	Presence	12 months	106 ± 138	226 ± 165	31 ± 33	33 ± 26
	Absence	12 months	84 ± 66	181 ± 111	27 ± 27	27 ± 25

## Appendix 4

Abundance of Collembola as affected by the presence of leaf litter species [*Cecropia andina* (CA), *Dictyocaryum lamarckianum* (DL), *Myrcia pubescens* (MP)*, Graffenrieda emarginata* (GE), *Cavendishia zamorensis* (CZ), and *Clusia* spp. (Cs)]. Boxplots show medians and quantiles of Collembola abundance for presence and absence of each leaf litter species. Violin plots illustrate kernel probability density. ****p* < .001; ***p* < .01.
