# Supplementary material for: Leaf litter identity rather than diversity shapes microbial functions and microarthropod abundance in tropical montane rainforests
Source: Ecol Evol. 2021 Jan 29;11(5):2360–74. doi: 10.1002/ece3.7208 (PMC7920764; doi:10.1002/ece3.7208)
Supplement: Supplementary file 1 — Supplementary Material [file ECE3-11-2360-s001.pdf]

RAW DATASET

P: *Dictyocaryum lamarckianum*
G: *Graffenrieda emarginata*
A: *Cecropia andina*
C: *Clusia* spp.
V: *Cavendishia zamorensis*
M: *Myrcia pubescens*

Date	Diversity	Block	Littertype	Mloss	Oribatida_ab	Collembola_ab	Prostigmata_ab	Mesostigmata_ab	BR	Cmic	Slope	qO2	C	N	C-toN
6 months	1	1	P	24,01	16	26	4	18	88,8566	6,1355	0,0040	14,4823	48,81871	0,856215	57,01688
6 months	1	2	P	29,54	56	22	10	28	121,5838	6,8422	0,0041	17,7697	49,339333	1,099444	44,87662
6 months	1	3	P	23,89	34	10	6	14	122,2446	7,8793	0,0018	15,5147	49,074875	1,24391	39,45211
6 months	1	4	P	26,31	4	8	6	24	109,3046	7,4537	0,0040	14,6645	49,376293	0,949986	51,97581
6 months	1	1	G	38,01	166	36	26	40	116,1864	10,8034	0,0049	10,7547	48,185871	1,089749	44,2174
6 months	1	2	G	38,89	34	30	12	8	110,4195	9,2846	0,0031	11,8928	47,505066	1,342642	35,38178
6 months	1	3	G	51,61	94	46	22	52	102,4573	9,5708	0,0050	10,7052	47,468388	1,061218	44,7301
6 months	1	1	A	27,68	18	42	20	22	81,6050	10,6535	0,0000	7,6599	42,137314	1,688045	24,9622
6 months	1	2	A	27,94	62	78	36	42	84,9011	7,4896	0,0000	11,3359	43,351112	1,404862	30,85791
6 months	1	3	A	28,87	50	48	32	64	88,6533	9,9545	0,0000	8,9059	41,795208	1,938895	21,5562
6 months	1	1	C	31,49	34	32	8	8	55,8632	5,2371	0,0082	10,6668	51,361385	0,747568	68,70463
6 months	1	2	C	30,97	118	58	28	12	56,2142	5,1650	0,0080	10,8836	49,337646	0,594274	83,02171
6 months	1	3	C	31,05	106	28	16	18	91,7219	6,3948	0,0073	14,3433	49,909821	0,627232	79,57155
6 months	1	1	V	27,77	54	18	8	16	100,7718	7,7420	0,0044	13,0162	49,843433	0,753191	66,17635
6 months	1	2	V	30,60	122	34	12	14	103,3333	8,7780	0,0040	11,7719	48,884365	1,041886	46,91911
6 months	1	3	V	29,85	16	4	12	4	118,6388	10,5590	0,0053	11,2358	49,419205	1,253915	39,41193
6 months	1	4	V	25,61	30	10	6	12	91,9918	7,0752	0,0000	13,0020	49,242031	0,794519	61,97716
6 months	1	1	M	19,55	128	14	4	8	95,4210	6,1242	0,0101	15,5810	45,993355	1,006596	45,69197
6 months	1	2	M	21,19	68	28	14	18	77,3440	5,2188	0,0059	14,8203	47,278255	0,852194	55,47828
6 months	1	3	M	27,16	32	6	6	6	103,9684	7,3870	0,0100	14,0745	46,400116	1,008363	46,01529
6 months	2	1	PG	41,02	22	34	26	4	128,3906	8,4370	0,0046	15,2177	48,5022905	0,972982	49,84911
6 months	2	2	PG	45,64	84	14	8	14	133,8526	8,4605	0,0000	15,8208	48,4221995	1,221043	39,65642
6 months	2	3	PG	40,21	82	48	30	58	139,6497	10,5170	0,0052	13,2785	48,2716315	1,152564	41,88195
6 months	2	1	PA	44,24	60	74	38	74	85,1699	6,2049	0,0024	13,7262	45,478012	1,27213	35,7495
6 months	2	2	PA	39,23	128	68	8	80	105,7843	8,1963	0,0065	12,9064	46,3452225	1,252153	37,01243
6 months	2	3	PA	47,43	44	50	24	4	104,1537	8,3616	0,0000	12,4562	45,4350415	1,5914025	28,55031
6 months	2	4	PA	42,50	40	14	32	30	81,6755	7,2549	0,0000	11,2579	44,8225305	1,5806195	28,35757
6 months	2	1	PC	43,40	100	12	10	18	107,1956	7,3322	0,0063	14,6199	50,0900475	0,8018915	62,46487
6 months	2	2	PC	40,76	58	10	0	4	117,6480	8,2305	0,0105	14,2941	49,3384895	0,846859	58,26057
6 months	2	3	PC	44,52	80	24	18	4	125,2117	7,7143	0,0034	16,2311	49,492348	0,935571	52,90069
6 months	2	1	PV	42,86	16	2	6	4	82,5981	6,6858	0,0053	12,3543	49,3310715	0,804703	61,30345
6 months	2	2	PV	35,63	132	18	10	18	89,9368	6,2222	0,0069	14,4542	49,111849	1,070665	45,87042
6 months	2	3	PV	41,31	124	74	26	10	95,1999	7,0063	0,0075	13,5877	49,24704	1,2489125	39,43194
6 months	2	4	PV	38,41	66	58	14	20	65,9041	5,2071	0,0044	12,6566	49,309162	0,8722525	56,53083
6 months	2	1	PM	39,11	108	14	26	42	107,9781	6,9210	0,0043	15,6015	47,4060325	0,9314055	50,89731
6 months	2	2	PM	37,15	44	26	8	36	105,5326	6,7777	0,0018	15,5706	48,308794	0,975819	49,5059
6 months	2	3	PM	45,48	68	6	20	6	101,6578	7,3070	0,0046	13,9125	47,7374955	1,1261365	42,39051
6 months	2	4	PM	40,91	10	20	2	4	72,9804	5,1276	0,0061	14,2330	47,714367	0,974802	48,94775
6 months	2	1	GA	46,03	42	114	42	42	122,3221	11,7956	0,0000	10,3702	45,1615925	1,388897	32,51616
6 months	2	2	GA	47,97	62	26	14	22	125,6293	10,4382	0,0000	12,0355	45,428089	1,373752	33,06862
6 months	2	3	GA	45,77	70	52	18	20	89,6495	9,9809	0,0096	8,9821	44,631798	1,5000565	29,75341
6 months	2	1	GC	42,71	190	16	6	24	96,8333	7,4755	0,0049	12,9534	49,773628	0,9186585	54,18077
6 months	2	2	GC	54,08	38	38	2	32	127,2667	7,9849	0,0047	15,9385	48,421356	0,968458	49,99841
6 months	2	3	GC	49,50	56	22	10	12	132,2394	9,6287	0,0050	13,7338	48,6891045	0,844225	57,67314
6 months	2	1	GV	42,56	70	26	4	20	76,6706	7,1511	0,0068	10,7216	49,014652	0,92147	53,1918
6 months	2	2	GV	46,19	48	6	8	16	146,0185	9,6307	0,0019	15,1617	48,1947155	1,192264	40,42286
6 months	2	3	GV	39,80	10	10	6	0	98,7586	7,6628	0,0020	12,8880	48,4437965	1,1575665	41,84969

6 months	2	4	GV	42,31	60	20	26	14	94,4866	7,4743	0,0044	12,6415	48,740587	0,8427385	57,83596
6 months	2	1	GM	45,76	116	24	2	20	119,5807	9,4706	0,0040	12,6265	47,089613	1,0481725	44,92544
6 months	2	2	GM	38,41	58	44	16	40	98,3231	6,8589	0,0017	14,3351	47,3916605	1,097418	43,18469
6 months	2	3	GM	42,01	32	12	14	16	114,9711	8,2447	0,0038	13,9449	46,934252	1,0347905	45,35628
6 months	2	1	AC	43,26	106	84	14	42	90,8808	9,1434	0,0085	9,9395	46,7493495	1,2178065	38,38816
6 months	2	2	AC	42,57	64	28	18	26	73,8590	5,9653	0,0215	12,3815	46,344379	0,999568	46,36441
6 months	2	3	AC	55,74	22	48	8	12	123,8083	12,9555	0,0000	9,5564	45,8525145	1,2830635	35,73675
6 months	2	1	AV	42,01	64	34	14	52	99,7509	10,1693	0,0000	9,8091	45,9903735	1,220618	37,67794
6 months	2	2	AV	45,64	14	12	2	18	110,5130	8,5518	0,0000	12,9227	46,1177385	1,223374	37,69717
6 months	2	3	AV	48,56	26	72	18	10	107,4440	11,1199	0,0000	9,6623	45,6072065	1,596405	28,56869
6 months	2	4	AV	40,76	28	6	14	12	92,0803	9,9697	0,0000	9,2360	44,7553995	1,502886	29,77964
6 months	2	1	AM	43,69	100	42	24	46	102,2721	7,7996	0,0081	13,1124	44,0653345	1,3473205	32,7059
6 months	2	2	AM	44,38	40	40	16	22	97,0872	8,5746	0,0036	11,3226	45,3146835	1,128528	40,1538
6 months	2	3	AM	44,66	86	14	24	32	105,7132	10,2516	0,0000	10,3118	44,097662	1,473629	29,92453
6 months	2	2	CV	41,60	112	34	2	22	111,3507	7,9659	0,0047	13,9785	49,1110055	0,81808	60,03203
6 months	2	3	CV	39,79	40	34	16	16	107,4496	8,4204	0,0048	12,7606	49,664513	0,9405735	52,80237
6 months	2	4	CV	42,99	30	6	8	0	94,0188	6,9133	0,0053	13,5997	49,8756465	0,7758205	64,28761
6 months	2	1	CM	41,17	76	26	14	28	84,7877	6,2702	0,0058	13,5224	48,67737	0,877082	55,49922
6 months	2	2	CM	44,10	52	32	10	16	75,4756	6,1584	0,0059	12,2556	48,3079505	0,723234	66,79436
6 months	2	3	CM	44,52	84	14	6	8	75,3917	6,4088	0,0026	11,7639	48,1549685	0,8177975	58,88373
6 months	2	1	VM	39,51	100	50	16	28	106,1839	7,2288	0,0043	14,6889	47,918394	0,8798935	54,45931
6 months	2	2	VM	43,12	10	32	6	10	76,1335	5,0821	0,0032	14,9807	48,08131	0,94704	50,77009
6 months	2	3	VM	39,67	46	16	6	2	130,1375	8,6746	0,0013	15,0021	47,9096605	1,131139	42,35524
6 months	2	4	VM	38,69	40	28	10	4	97,8557	6,3492	0,0036	15,4122	47,647236	0,8970685	53,11438
6 months	4	1	PGAC	46,03	32	36	10	4	110,7919	8,3250	0,0045	13,3083	47,62582	1,09539425	43,47825
6 months	4	2	PGAC	45,45	172	40	20	8	89,7162	6,8972	0,0042	13,0077	47,3832893	1,1103055	42,6759
6 months	4	3	PGAC	46,87	42	46	32	26	134,4515	8,4107	0,0084	15,9858	47,062073	1,21781375	38,64472
6 months	4	4	PGAC	39,53	20	28	36	34	83,6225	7,8303	0,0004	10,6793	47,0983665	1,20232975	39,17259
6 months	4	1	PGAV	45,10	30	14	8	20	99,9570	7,0032	0,0013	14,2730	47,246332	1,0968	43,07652
6 months	4	2	PGAV	39,94	100	14	14	30	106,3897	8,7103	0,0011	12,2142	47,269969	1,2222085	38,67586
6 months	4	3	PGAV	47,00	54	18	30	14	132,6853	10,3179	0,0050	12,8598	46,939419	1,3744845	34,15056
6 months	4	4	PGAV	43,97	32	32	8	16	141,3329	10,1003	0,0007	13,9929	46,7815588	1,211679	38,60887
6 months	4	2	PGAM	40,62	62	28	0	14	95,7062	7,0050	0,0003	13,6626	46,8684415	1,1747855	39,89532
6 months	4	3	PGAM	42,29	62	62	72	64	86,4958	8,5815	0,0019	10,0794	46,1846468	1,3130965	35,17232
6 months	4	4	PGAM	43,46	68	48	16	16	103,6561	6,7599	0,0052	15,3339	45,9841613	1,26295375	36,41001
6 months	4	1	PGCV	42,15	98	20	8	36	100,8681	7,0527	0,0063	14,3021	49,5523498	0,86168075	57,50662
6 months	4	2	PGCV	50,61	78	46	14	20	129,1981	8,0189	0,0065	16,1117	48,7666025	1,0195615	47,83096
6 months	4	3	PGCV	47,56	4	4	0	2	99,5325	7,3952	0,0053	13,4590	48,9680723	1,04656875	46,78916
6 months	4	4	PGCV	54,64	22	10	0	34	73,4231	4,7553	0,0164	15,4404	49,3416823	0,84814625	58,17591
6 months	4	1	PGCM	42,57	94	10	30	24	52,5946	5,2480	0,0046	10,0217	48,5898303	0,925032	52,52773
6 months	4	2	PGCM	44,93	58	24	6	12	86,1923	5,2364	0,0041	16,4601	48,365075	0,9721385	49,75122
6 months	4	3	PGCM	42,62	68	22	44	8	84,0081	6,8826	0,0068	12,2058	48,2133	0,98518075	48,93853
6 months	4	4	PGCM	37,24	64	62	12	40	110,7332	51,7453	0,0000	2,1400	48,5442848	0,899421	53,97282
6 months	4	1	PGVM	42,29	132	104	12	32	88,0436	7,7962	0,0015	11,2932	48,2103423	0,92643775	52,0384
6 months	4	2	PGVM	41,86	166	18	6	40	94,1381	7,1445	0,0046	13,1764	48,2517548	1,0840415	44,51098
6 months	4	3	PGVM	45,47	52	24	46	40	95,8897	7,6093	0,0025	12,6017	48,090646	1,1418515	42,11638
6 months	4	4	PGVM	42,01	62	32	8	26	88,4024	6,9190	0,0030	12,7767	48,227477	0,90877025	53,06894
6 months	4	1	PACV	45,35	134	34	10	18	122,0811	10,0135	0,0055	12,1916	47,246332	1,0968	43,07652
6 months	4	2	PACV	46,45	110	24	12	38	100,0649	7,3593	0,0056	13,5970	47,269969	1,2222085	38,67586
6 months	4	3	PACV	44,10	62	12	30	20	119,4160	10,9446	0,0012	10,9109	46,939419	1,3744845	34,15056
6 months	4	4	PACV	47,98	66	54	30	54	160,1359	9,3592	0,0032	17,1101	46,7815588	1,211679	38,60887
6 months	4	1	PACM	42,31	66	26	4	16	91,7155	6,3372	0,0065	14,4726	47,077691	1,074606	43,80926
6 months	4	2	PACM	41,20	42	22	4	30	96,1633	5,9056	0,0204	16,2833	47,3265865	0,9876935	47,91627

6 months	4	3	PACM	47,55	42	10	8	6	92,7620	6,9594	0,0063	13,3291	46,795005	1,2046	38,84692
6 months	4	4	PACM	40,05	22	14	14	16	117,7666	8,6271	0,0041	13,6508	46,551691	1,22949475	37,86246
6 months	4	1	PAVM	49,94	32	28	14	18	90,7812	7,3593	0,0045	12,3355	46,698203	1,07601175	43,39934
6 months	4	2	PAVM	43,97	28	28	12	4	126,7833	8,0413	0,0017	15,7666	47,2132663	1,0995965	42,9369
6 months	4	2	PAVM	42,31	34	30	6	10	105,0309	8,7222	0,0049	12,0418	46,672351	1,36127075	34,28587
6 months	4	4	PAVM	41,05	32	60	24	26	107,6467	8,9487	0,0022	12,0293	46,2348833	1,238844	37,32099
6 months	4	1	PCVM	46,19	38	34	30	40	98,1478	8,7158	0,0029	11,2610	49,0042208	0,8408925	58,27644
6 months	4	2	PCVM	40,91	26	12	0	8	98,8207	6,6018	0,0038	14,9689	48,7098998	0,8969495	54,30618
6 months	4	3	PCVM	42,99	54	40	16	18	110,7844	7,5048	0,0048	14,7619	48,7010043	1,033355	47,12902
6 months	4	4	PCVM	41,60	34	32	0	14	98,4518	5,9988	0,0042	16,4120	48,7950068	0,87531125	55,74589
6 months	4	1	GACV	52,55	102	40	8	38	92,1893	6,8547	0,0060	13,4491	47,8820008	1,06963825	44,76467
6 months	4	2	GACV	48,26	80	46	26	32	71,4833	6,4790	0,0027	11,0330	47,2695473	1,095916	43,13245
6 months	4	3	GACV	53,26	86	66	22	16	93,5283	7,7581	0,0053	12,0555	47,1481555	1,220315	38,63605
6 months	4	4	GACV	44,23	26	16	22	20	131,9321	11,6759	0,0107	11,2996	47,064801	1,163463	40,45234
6 months	4	1	GACM	51,30	134	64	8	38	70,6095	6,5174	0,0073	10,8340	46,8680198	1,048493	44,70036
6 months	4	2	GACM	44,11	58	10	0	16	85,3421	7,2484	0,0000	11,7739	46,3933833	1,158927	40,03132
6 months	4	3	GACM	51,73	58	12	14	6	119,8813	11,2739	0,0001	10,6336	46,2674035	1,21473775	38,08839
6 months	4	1	GAVM	45,50	56	42	26	46	103,5194	8,8639	0,0050	11,6788	46,5399933	1,13439525	41,02626
6 months	4	2	GAVM	48,68	16	18	2	2	101,2582	8,6127	0,0018	11,7569	46,7546995	1,160396	40,29202
6 months	4	3	GAVM	43,42	72	20	18	10	138,2529	11,4669	0,0000	12,0567	46,2707293	1,31559775	35,17088
6 months	4	4	GAVM	46,31	18	60	26	6	140,3522	9,3019	0,0021	15,0885	45,9505958	1,224087	37,53867
6 months	4	1	GCVM	41,04	56	18	0	16	93,9380	8,7627	0,0098	10,7202	48,846011	0,899276	54,31704
6 months	4	2	GCVM	44,92	66	40	12	8	109,7982	7,6563	0,0022	14,3408	48,251333	0,957749	50,37994
6 months	4	3	GCVM	41,19	74	32	32	32	115,1705	9,1050	0,0059	12,6491	48,2993825	0,987682	48,90175
6 months	4	4	GCVM	46,31	40	40	6	16	103,1588	6,5416	0,0000	15,7696	48,5107193	0,86055425	56,37148
6 months	4	1	ACVM	47,68	28	12	6	10	75,1851	8,6974	0,0015	8,6446	47,3338718	1,04885	45,12931
6 months	4	3	ACVM	46,73	100	32	24	14	98,1635	7,8193	0,0039	12,5540	47,2128445	0,973304	48,50781
6 months	4	3	ACVM	44,38	48	28	46	14	88,1036	7,4665	0,0079	11,7998	46,8810875	1,20710125	38,83774
6 months	4	4	ACVM	45,06	26	16	28	20	87,2977	7,7166	0,0032	11,3130	46,5181255	1,190628	39,07024
12 months	1	1	P	62,90	140	14	6	24	227,7110	15,2443	0,0154	14,9374	47,809055	1,328671	35,98261
12 months	1	2	P	62,92	58	8	6	10	332,6587	19,1576	0,0550	17,3643	48,560013	1,169437	41,52427
12 months	1	3	P	52,04	64	24	2	16	199,9408	9,9331	0,0009	20,1288	48,164043	1,334355	36,09537
12 months	1	4	P	54,03	56	10	14	4	283,3857	15,6018	0,0084	18,1637	47,981323	1,094235	43,84919
12 months	1	1	G	59,04	172	84	16	50	108,7621	13,4921	0,0449	8,0612	46,835678	1,376157	34,03367
12 months	1	2	G	54,75	40	16	6	0	270,1823	18,3983	0,0207	14,6852	49,544209	1,424384	34,7829
12 months	1	3	G	59,51	80	124	18	12	178,1733	10,9507	0,0108	16,2705	46,951397	1,199822	39,13197
12 months	1	1	A	46,67	164	140	30	18	155,3828	29,1968	0,0452	5,3219	42,408607	1,654239	25,63632
12 months	1	2	A	43,21	60	80	24	2	123,5496	21,9656	0,1059	5,6247	42,980858	1,496193	28,72681
12 months	1	3	A	59,76	56	76	4	8	582,8309	47,9980	0,0258	12,1428	41,898041	2,285372	18,33314
12 months	1	1	C	56,82	64	16	8	4	169,7293	23,1463	-0,0035	7,3329	49,066624	0,711896	68,92387
12 months	1	2	C	47,95	60	20	0	0	173,8224	23,0025	-0,0081	7,5567	48,88826	0,763915	63,99699
12 months	1	3	C	62,55	272	176	40	26	141,7073	22,2575	-0,0079	6,3667	49,50312	0,715545	69,1824
12 months	1	1	V	50,26	74	56	6	24	112,3958	7,4104	0,0043	15,1672	49,53664	1,079651	45,88209
12 months	1	2	V	50,47	166	38	38	22	232,1535	16,1760	0,0030	14,3518	49,991589	1,116135	44,78991
12 months	1	3	V	55,68	146	58	20	18	323,4665	8,6018	-0,0004	37,6044	49,298641	1,036091	47,58138
12 months	1	4	V	49,31	38	28	0	6	187,1569	34,9929	-0,0060	5,3484	49,379135	1,086113	45,46409
12 months	1	1	M	45,08	184	48	10	4	133,2724	8,2568	-0,0046	16,1409	45,90731	1,032137	44,47792
12 months	1	2	M	46,20	76	100	14	2	134,5101	18,6017	0,0293	7,2310	46,377983	1,027826	45,12241
12 months	1	3	M	53,86	60	88	18	6	190,4329	11,8514	0,0257	16,0684	45,437653	0,988101	45,98483
12 months	2	1	PG	63,99	182	44	16	14	186,8789	21,1181	0,0377	8,8492	47,3223665	1,352414	34,99104
12 months	2	2	PG	60,07	48	42	12	2	542,8241	43,8582	0,0379	12,3768	49,052111	1,2969105	37,82228
12 months	2	3	PG	58,76	202	34	44	70	183,4589	12,7485	0,0127	14,3907	47,55772	1,2670885	37,53307
12 months	2	1	PA	51,49	268	92	48	18	158,2733	11,9009	0,0309	13,2993	45,108831	1,491455	30,24485

12 months	2	2	PA	53,16	82	18	4	16	172,4296	11,0390	0,0101	15,6200	45,7704355	1,332815	34,34118
12 months	2	3	PA	57,68	166	60	32	36	398,7255	24,3868	0,0258	16,3500	45,031042	1,8098635	24,88091
12 months	2	4	PA	58,53	74	16	32	30	366,3067	18,6633	0,0068	19,6271	45,68787	1,36809	33,39537
12 months	2	1	PC	50,15	166	64	44	22	323,1253	14,3375	0,0232	22,5371	48,4378395	1,0202835	47,47488
12 months	2	2	PC	58,61	138	18	12	6	185,5294	18,3983	0,0445	10,0840	48,7241365	0,966676	50,40379
12 months	2	3	PC	47,82	36	6	0	6	430,3704	13,7987	0,0145	31,1891	48,8335815	1,02495	47,64484
12 months	2	1	PV	56,44	14	2	8	2	127,9167	7,8027	0,0023	16,3940	48,6728475	1,204161	40,42055
12 months	2	2	PV	56,06	62	26	16	4	99,1970	6,3492	-0,0005	15,6235	49,275801	1,142786	43,11901
12 months	2	3	PV	57,73	182	16	20	38	225,0352	12,8011	0,0004	17,5794	48,731342	1,185223	41,11576
12 months	2	4	PV	59,69	58	16	6	2	107,4961	4,6906	-0,0046	22,9175	48,680229	1,090174	44,65363
12 months	2	1	PM	49,65	190	28	16	22	146,9961	13,4417	0,0195	10,9358	46,8581825	1,180404	39,69673
12 months	2	2	PM	58,45	80	28	12	20	239,0430	17,1718	0,0109	13,9207	47,468998	1,0986315	43,20739
12 months	2	3	PM	57,69	48	8	8	0	509,7032	40,6197	0,0202	12,5482	46,800848	1,161228	40,30289
12 months	2	4	PM	52,64	54	24	6	10	303,7629	20,9020	0,0545	14,5327	47,157091	1,0998305	42,87669
12 months	2	1	GA	48,90	196	192	42	30	78,7388	6,7844	0,0043	11,6059	44,6221425	1,515198	29,44971
12 months	2	2	GA	54,47	48	48	10	4	82,6993	8,3726	0,0244	9,8774	46,2625335	1,4602885	31,68041
12 months	2	3	GA	53,94	108	140	8	18	73,9922	7,3593	0,0107	10,0542	44,424719	1,742597	25,4934
12 months	2	1	GC	46,28	128	0	0	0	139,3357	8,7459	0,0020	15,9316	47,951151	1,0440265	45,92906
12 months	2	2	GC	57,06	116	68	10	20	109,3963	5,7823	-0,0041	18,9191	49,2162345	1,0941495	44,98127
12 months	2	3	GC	52,61	56	44	24	2	112,0754	7,0387	0,0006	15,9228	48,2272585	0,9576835	50,35824
12 months	2	1	GV	48,60	78	20	8	34	113,2169	6,8660	0,0034	16,4896	48,186159	1,227904	39,24261
12 months	2	2	GV	57,07	198	42	48	26	113,6693	8,6144	0,0028	13,1952	49,767899	1,2702595	39,17932
12 months	2	3	GV	52,66	36	6	6	4	263,8762	6,8227	0,0087	38,6761	48,125019	1,1179565	43,04731
12 months	2	4	GV	52,21	52	20	8	10	564,7423	16,3878	0,0168	34,4612	48,5974235	1,079735	45,00866
12 months	2	1	GM	54,70	200	136	50	44	86,0311	6,5847	0,0018	13,0654	46,371494	1,204147	38,50983
12 months	2	2	GM	50,90	152	80	18	14	84,0236	5,8769	-0,0034	14,2973	47,961096	1,226105	39,11663
12 months	2	3	GM	52,08	60	52	14	16	118,5714	5,1515	0,0126	23,0167	46,194525	1,0939615	42,22683
12 months	2	1	AC	53,38	108	76	24	20	212,5202	13,2349	0,0106	16,0575	45,7376155	1,1830675	38,66019
12 months	2	2	AC	76,54	160	80	20	8	223,1183	14,4813	0,0159	15,4074	45,934559	1,130054	40,64811
12 months	2	3	AC	46,09	24	156	14	8	306,6045	12,4120	0,0072	24,7022	45,7005805	1,5004585	30,45774
12 months	2	1	AV	46,70	70	44	32	14	110,3775	7,0602	0,0057	15,6338	45,9726235	1,366945	33,63166
12 months	2	2	AV	48,60	108	16	2	8	115,7924	9,7166	-0,0075	11,9169	46,4862235	1,306164	35,58988
12 months	2	3	AV	67,63	6	24	8	8	108,2441	10,7591	-0,0128	10,0607	45,598341	1,6607315	27,45678
12 months	2	4	AV	49,68	78	22	0	12	98,5625	15,5006	-0,0366	6,3586	46,386776	1,364029	34,00718
12 months	2	1	AM	51,98	92	44	6	14	89,6605	7,9045	0,0006	11,3430	44,1579585	1,343188	32,87549
12 months	2	2	AM	53,57	52	16	2	0	127,5741	9,3218	0,0025	13,6855	44,6794205	1,2620095	35,40339
12 months	2	3	AM	57,48	16	92	10	10	112,8599	9,9490	0,0069	11,3439	43,667847	1,6367365	26,67983
12 months	2	2	CV	53,36	72	26	6	8	105,7364	8,0422	0,0068	13,1478	49,4399245	0,940025	52,59427
12 months	2	3	CV	58,24	116	68	56	50	207,9521	17,7009	0,0102	11,7481	49,4008805	0,875818	56,40542
12 months	2	4	CV	63,67	172	34	14	6	466,1271	11,3428	0,0184	41,0944	49,4438685	0,920928	53,68918
12 months	2	1	CM	51,90	124	136	16	14	298,2929	13,6078	0,0092	21,9207	47,486967	0,8720165	54,4565
12 months	2	2	CM	52,63	12	12	2	0	313,7008	11,8213	0,0064	26,5369	47,6331215	0,8958705	53,16965
12 months	2	3	CM	57,64	408	156	30	80	111,7857	6,8273	0,0068	16,3733	47,4703865	0,851823	55,72799
12 months	2	1	VM	55,29	116	52	2	22	138,1970	9,0342	-0,0057	15,2971	47,721975	1,055894	45,1958
12 months	2	2	VM	53,72	90	44	18	6	95,7692	5,9913	-0,0054	15,9848	48,184786	1,0719805	44,94931
12 months	2	3	VM	55,22	164	62	8	24	291,0220	15,4824	0,0006	18,7970	47,368147	1,012096	46,80203
12 months	2	4	VM	55,88	64	12	10	8	110,0440	88,2112	0,0004	1,2475	47,855997	1,0957695	43,67342
12 months	4	1	PGAC	40,15	214	82	26	14	374,7029	13,7588	0,0178	27,2338	46,529991	1,26774075	36,70308
12 months	4	2	PGAC	28,65	76	48	8	20	97,5137	7,3593	0,0063	13,2503	47,493335	1,21348225	39,13805
12 months	4	3	PGAC	59,00	148	74	40	28	177,6132	7,2685	0,0011	24,4361	46,6291503	1,3837735	33,6971
12 months	4	4	PGAC	36,98	90	52	18	2	93,5031	6,8142	0,0033	13,7218	47,1750135	1,14132	41,33373
12 months	4	1	PGAV	51,15	92	24	2	0	217,1308	13,0884	0,0072	16,5895	46,647495	1,3596795	34,30771
12 months	4	2	PGAV	51,23	148	42	8	16	305,7895	14,2539	0,0865	21,4531	47,7691673	1,30153725	36,70211

12 months	4	3	PGAV	35,95	200	70	32	6	146,0185	12,6985	0,0077	11,4989	46,5780305	1,46391	31,81755
12 months	4	4	PGAV	45,44	86	36	12	4	118,4948	7,4734	0,0013	15,8555	47,1426468	1,2239125	38,51799
12 months	4	2	PGAM	53,88	40	18	12	16	134,5606	9,3664	0,0080	14,3663	46,8657658	1,27946	36,62933
12 months	4	3	PGAM	48,13	20	40	4	2	135,8811	13,1609	0,0120	10,3246	45,6127835	1,4519125	31,41566
12 months	4	4	PGAM	50,28	154	84	14	48	203,8976	18,5900	0,0180	10,9681	46,3810778	1,22874075	37,74684
12 months	4	1	PGCV	41,54	146	48	16	8	130,4286	8,0427	0,0105	16,2170	48,3119993	1,12409375	42,97862
12 months	4	2	PGCV	46,90	96	24	10	6	156,4484	7,0089	0,0019	22,3214	49,2460178	1,11846775	44,0299
12 months	4	3	PGCV	45,44	78	58	6	6	65,3002	9,0049	0,0090	7,2516	48,4793003	1,07145325	45,2463
12 months	4	4	PGCV	41,84	108	6	0	10	72,2117	7,5445	0,0004	9,5715	48,671193	1,002362	48,5565
12 months	4	1	PGCM	58,13	180	32	12	38	101,2500	17,0683	-0,0213	5,9320	47,4046668	1,11221525	42,62185
12 months	4	2	PGCM	55,11	246	54	28	42	50,0441	5,3247	0,0084	9,3985	48,3426163	1,0963905	44,09252
12 months	4	3	PGCM	49,86	208	58	14	22	226,8925	13,8245	0,0097	16,4123	47,5140533	1,05945575	44,84761
12 months	4	4	PGCM	43,88	84	12	4	8	126,3564	6,7646	-0,0115	18,6790	47,909624	1,00719025	47,5676
12 months	4	1	PGVM	58,30	194	100	64	42	123,8566	9,2420	0,0039	13,4016	47,5221708	1,204154	39,46519
12 months	4	2	PGVM	55,33	96	20	0	8	262,0862	13,1032	0,0297	20,0017	48,6184485	1,1844455	41,04743
12 months	4	3	PGVM	54,57	130	34	8	8	104,7573	4,5013	0,0029	23,2725	47,4629335	1,13959225	41,64905
12 months	4	4	PGVM	50,69	12	32	6	0	122,0588	11,3998	0,0362	10,7071	47,8772573	1,08978275	43,93285
12 months	4	1	PACV	49,36	108	38	10	8	115,6002	8,5430	0,0061	13,5316	46,647495	1,3596795	34,30771
12 months	4	2	PACV	49,78	114	0	10	10	133,3533	8,9489	-0,0002	14,9016	47,7691673	1,30153725	36,70211
12 months	4	3	PACV	68,35	34	38	14	4	210,9583	15,3166	0,0233	13,7732	46,5780305	1,46391	31,81755
12 months	4	4	PACV	52,18	88	54	34	44	254,4345	11,2142	0,0295	22,6886	47,1426468	1,2239125	38,51799
12 months	4	1	PACM	46,24	96	24	8	12	137,8813	10,6301	0,0081	12,9708	46,297899	1,18173575	39,17788
12 months	4	2	PACM	57,40	248	38	6	16	82,8653	5,1850	0,0014	15,9818	46,7017785	1,11434275	41,90971
12 months	4	3	PACM	57,85	62	26	14	16	305,2355	24,5311	0,0472	12,4428	46,2507143	1,33084325	34,75294
12 months	4	4	PACM	36,27	6	0	12	2	140,4615	12,0014	0,0075	11,7038	46,8043003	1,14933725	40,72286
12 months	4	1	PAVM	46,02	90	50	6	2	243,6919	19,2541	0,0108	12,6566	46,415403	1,2736745	36,44212
12 months	4	2	PAVM	56,12	88	34	16	8	284,4479	13,0628	0,0218	21,7754	46,9776108	1,20239775	39,06994
12 months	4	2	PAVM	39,39	142	58	26	12	104,0100	6,3910	0,0021	16,2745	46,1995945	1,41097975	32,74292
12 months	4	4	PAVM	61,06	28	44	0	12	131,8992	5,2200	0,0085	25,2681	46,7719335	1,23192975	37,9664
12 months	4	1	PCVM	57,50	52	12	12	0	104,3788	7,3593	0,0426	14,1832	48,0799073	1,03808875	46,3158
12 months	4	2	PCVM	54,56	90	6	2	4	136,6995	8,7501	0,0028	15,6227	48,4544613	1,01932825	47,53568
12 months	4	3	PCVM	58,76	26	4	10	2	127,2514	11,3439	0,0096	11,2176	48,1008643	1,018523	47,2261
12 months	4	4	PCVM	51,96	62	8	6	4	183,7350	8,8689	0,0273	20,7167	48,3004798	1,01037925	47,80431
12 months	4	1	GACV	48,62	108	56	16	6	565,5993	29,3104	0,0284	19,2969	46,9618873	1,20548575	38,95682
12 months	4	2	GACV	47,07	54	46	18	6	66,5664	5,3120	0,0033	12,5313	47,851229	1,20015675	39,87082
12 months	4	3	GACV	60,89	248	46	40	10	499,1957	34,7558	0,0252	14,3629	46,9127998	1,3092075	35,83298
12 months	4	4	GACV	58,51	146	52	30	14	202,9074	24,9284	0,0329	8,1396	47,5244665	1,1392895	41,71413
12 months	4	1	GACM	49,71	312	528	28	70	149,7816	15,3785	0,0115	9,7397	46,9478275	1,1780795	39,85115
12 months	4	2	GACM	57,42	40	36	4	0	131,3585	9,2146	0,0022	14,2554	45,9475528	1,29721	35,42029
12 months	4	3	GACM	58,63	256	224	34	78	174,5252	15,0182	0,0264	11,6209	46,7628975	1,14411775	40,87245
12 months	4	1	GAVM	49,97	196	50	18	14	209,3694	10,6080	0,0065	19,7368	46,1720588	1,285546	35,9163
12 months	4	2	GAVM	60,04	14	10	2	2	339,2460	13,3169	0,0127	25,4749	47,2236598	1,2661345	37,29751
12 months	4	3	GAVM	38,28	98	10	10	6	150,6048	9,3179	0,0050	16,1630	45,896433	1,3773465	33,32236
12 months	4	4	GAVM	57,88	114	14	6	20	233,0734	8,0102	0,0039	29,0972	46,7305308	1,22671025	38,09419
12 months	4	1	GCVM	49,30	114	78	20	16	89,1330	6,6488	0,0108	13,4059	47,836563	1,04996025	45,56036
12 months	4	2	GCVM	37,65	176	40	38	10	137,7196	7,3136	0,0110	18,8306	48,7005103	1,083065	44,96545
12 months	4	3	GCVM	44,81	178	38	34	34	121,2063	6,6234	0,0038	18,2997	47,7977028	0,98488975	48,53102
12 months	4	4	GCVM	48,50	134	50	18	20	120,8516	7,1895	0,0017	16,8095	48,259077	1,00515975	48,01135
12 months	4	1	ACVM	54,38	56	20	14	10	192,4252	15,7093	0,0150	12,2491	46,7297953	1,11948075	41,74238
12 months	4	3	ACVM	55,64	26	14	0	6	88,0896	5,3459	0,0002	16,4779	47,0596725	1,10101725	42,74199
12 months	4	3	ACVM	54,29	164	70	40	34	214,9107	15,7700	0,0104	13,6278	46,5343638	1,25627725	37,04148
12 months	4	4	ACVM	35,07	108	34	32	24	175,5322	15,3989	0,0233	11,3990	47,1537533	1,14730675	41,09952
